# Everybody's Page

**Published:** 1889-03-23

**Authors:** 


### Everybody's Page.

The annotation lastweek, entitled "From the Court to the Hospital," was founded on a suggestion made by the Rev. F. O. Morris, the eminent ornithologist. His successful labours in the cause of humanity are well known, and we regret that by inadvertence the source of the kindly suggestion should have been omitted.

Southern California has been nick-named "the one-lung county" since it became a favourite resort for consumptives.

During the year 1888 there were 12,720,000 bottles of Apollinaris water put up at the Apollinaris spring. It was this water, we believe, of which the negro in search of a definition said, "It tastes like as if your foot's asleep."

The Minister of Public Instruction in France has issued a regulation that every child who has attained the age of ten must be re-vaccinated by the medical officer attached to the school before he can be admitted.

A short time ago a lady, the first of her sex, graduated in medicine in Mexico. As an appropriate compliment, her fellow students of the ungentle sex got up an amateur bull fight in honour of the occasion.

M. Leven recently expressed to the Société de Biologie the opinion that obesity is a nervous disorder. He advised as treatment the avoidance of mental and physical fatigue, and a diet of eggs, milk, rice, and potatoes. We should like to know the facts on which M. Leven bases his theory.

Bacteriology has taught our surgeons such care of their hands and instruments in their endeavours to prevent septic infection that a new proverb has been invented for their benefit: "The fear of the microbe is the beginning of cleanliness."

Three French doctors, M. Brown-Séquard, M. Arsouval, and M. R. Wurtz, have almost simultaneously discovered that air which has been expired contains a volatile poisonous alkaloid, identical with an alkaloid circulating in the blood. It is thought that one cause of phthisis may be that this poison is not perfectly got rid of by the weaker action of feeble lungs. Retained in the system it becomes a source of infection.

Celery acts upon the nervous system, and is a cure for rheumatism and neuralgia. Tomatos stimulate the liver, and spinach and the common dandelion, prepared in the same way, have a direct effect on diseases of the kidney. Onions, garlic, and olives promote digestion, by stimulating the circulatory system, with the consequent increase of the saliva and gastric juice. Raw onions are also regarded as a remedy for sleeplessness, and the French believe that onion soup is an excellent tonic in cases of debility of the digestive organs.

Among disinfectants the onion has not received the honour which is its due. The onion is a very sensitive organism, and takes everything in the way of disease that comes in its way, thus acting as a protection to human beings in its neighbourhood. During our last epidemic of cholera it was a puzzle to the sanitary inspectors of a northern town why the inhabitants of one cottage in a row were not touched by the disease which was raging among their neighbours. Finally some one noticed a net of onions hanging up in the fortunate house, and on examination these proved all to have become diseased. They absolutely seemed to have absorbed the infection. Of course this useful quality in our much maligned vegetable has a dark side. It is dangerous to eat an onion which shows symptoms of decay; one cannot tell what may have caused the disease.

Race has a marked effect in determining forms of insanity. The Teutonic and Scandinavian races are more subject to the morbid and melancholy forms of mental disease, while the excitable Celts are more liable to acute mania.

Medical practice in the remoter parts of America has its disadvantages. In Georgia an infuriated father, whose child had died of typhoid fever, killed Dr. J. B. Manson, the physician who had been in attendance.

An American fell ill in Paris, and was told that his end was near. He immediately ordered the following despatch to be sent by cable as soon as the breath was out of his body: "Am dead, corpse will follow by next steamer."

A man who died in Constantinople last year, named Dimitrios Antippa, had attained the age of a hundred and fifteen years. He was a native of Cephalonia, where he was born in 1772, and passed part of his early life in Paris. He was there during the Reign of Terror, and numbered Marat, Danton, and Robespierre among his personal friends.

Quackery is said to be much more rampant at the Antipodes than here. There is, indeed, a law regulating the practise of medicine; but the Chief Secretary of Victoria is rather favourable to unqualified practitioners, and will not authorise police proceedings being taken against them. In view of this happy hunting-ground, we may hope for a speedy emigration of the quacks of England.

There is a great deposit of nitrate of soda, which when purified is saltpetre, along the Peruvian coast. It stretches for hundreds of miles, and now that saltpetre is a component of so many chemicals, it is of incalculable value. Before its value was fully known, some shrewd men chose "claims" from it, after the fashion of the Californian miners. The Government has now prevented this method of appropriation, but not before a few millionaire fortunes have been founded on it.

If the condensed breath collected on the windows or walls of a room that several people have been occupying were assembled and burned, it would give off a smell like singed hair, thus showing the presence of organic matter. If it were left for a few days, and then examined under the microscope, it would be found to be putrescent, and alive with animalculæ. In such a condition it would form a powerful factor in promoting disease; but, of course, it can always be got rid of by the free circulation of fresh air through the room.

Is this following profanity or merely insanity? It is the prayer of a "Christian Scientist" for a dyspeptic patient: "Holy Reality, we believe that Thou art everywhere present; we believe that Thou art in this patient's stomach, in every fibre, in every cell, in every atom; that Thou art the sole only Reality of that stomach. Forgive us our sins in that we have this day talked about our back-aches, that we have told our neighbours that our food hurts us, that we mentioned to a visitor that there was a lump in our stomach, that we wasted our valuable time, which should have been spent in Thy service, in worrying for fear our stomach should grow worse, in that we have disobeyed Thy blessed law in thinking that some kind of medicine would help us. Help us to believe that all evil is utterly unreal, that it is atheism and denial of God to say, 'I am ill.' Help us to stoutly affirm, with our eyes fixed on Thee, that we have no dyspepsia, that we never had dyspepsia, that we will never have dyspepsia, that there is no such thing, that there never was any such thing, that there never will be any such thing. Amen."

